# The burden of somatic comorbidities in patients surviving a traumatic brain injury

**DOI:** 10.1007/s00701-025-06617-1

**Published:** 2025-08-07

**Authors:** Christian Mirian, Therese Ovesen, Lasse Rehné Jensen, Thomas Scheike, Jacob Bertram Springborg

**Affiliations:** 1 Department of Neurosurgery, Copenhagen University Hospital, Copenhagen, Denmark; 2 Department of Otorhinolaryngology, Head and Neck Surgery, Gødstrup Hospital, Herning, Denmark; 3 Department of Clinical Medicine, Aarhus University, Aarhus, Denmark; 4 Section of Biostatistics, Department of Public Health, University of Copenhagen, Copenhagen, Denmark

**Keywords:** TBI, Traumatic brain injury, Comorbidity, Intracerebral hemorrhage, Stroke, Cerebrovascular

## Abstract

**Background:**

The long-term development of somatic comorbidities in patients surviving a traumatic brain injury (TBI) may contribute to both the individual and public health burden; however, a systematic investigation has not yet been undertaken.

**Methods:**

We investigated the long-term burden of somatic comorbidities in patients surviving a TBI. We included all Danish residents (≥ 18.0 years) who were hospitalized with a TBI between 1994 and 2018 and survived beyond discharge (*n* = 153,177). TBIs were defined within a selected list of ICD-10 codes. Each TBI patient was age-matched to five non-TBI Controls (“Controls”, *n* = 752,224). The following age groups were considered: 18.0–39.9, 40.0–69.9, and ≥ 70.0. Within each age group, we analyzed (1) TBI patients *vs* Controls and (2) subsequently categorized patients based on their length of stay (LOS) to better capture that patients with more severe injuries stay longer in the hospital (*vs* Controls).

Somatic comorbidities within the Charlson Comorbidity Index (CCI) were considered. Adjusted odds ratios for developing cerebrovascular disease, cancer, diabetes, and cardiovascular disease were obtained using binomial regression. Using all CCI conditions, we employed a recurrent event regression weighted by the corresponding CCI scores to estimate the adjusted mean cumulative CCI score since index.

**Results:**

The landscape of CCI conditions shifts in TBI survivors and is predominated by a higher long-term odds ratio of developing cerebrovascular disease, especially non-traumatic intracerebral hemorrhage (ICH), even 10 years post-index; the risk is correlated with LOS. Cancer risk is significantly lower in TBI survivors. Irrespective of age group, the mean cumulative CCI score is significantly higher in TBI survivors.

**Conclusion:**

The indirect consequences of surviving a TBI extend far beyond the immediate post-index period by exacerbating functional decline in survivors through the compounding effect of somatic comorbidities that cumulatively affect the capacity to function physically, cognitively, or socially. TBI emerge as an independent risk factor for developing ICH.

**Supplementary information:**

The online version contains supplementary material available at 10.1007/s00701-025-06617-1.

## Introduction

Traumatic brain injuries (TBIs) contribute significantly to the global burden of disease, particularly when measured in years lived with TBI-induced disability (YLD) [23, 30]. However, the indirect consequences of TBIs, such as an increased susceptibility to developing somatic comorbidities, remain insufficiently investigated despite the potential major contribution to both the individual and public health burden.

TBI survivors have a significantly higher risk of developing a range of somatic conditions, such as metabolic disorders, epilepsy, stroke, cardiovascular disease, and dementia [1, 5, 7, 8, 10, 11, 16, 17, 21, 22]. However, most previous research has focused on the association between TBIs and the risk of developing psychiatric disorders or lacks the scope of a nationwide TBI cohort design, often limited by small cohort sizes, population-based settings, and short follow-up periods [24, 40, 43]. Managing the presence and severity of accumulated somatic comorbidities may be critical for effective long-term rehabilitation of TBI survivors, particularly for their reintegration into society [9, 17, 18, 27, 31]. Thus, a better understanding of secondary health effects from TBIs is essential for establishing and implementing targeted interventions to mitigate the potential decline of somatic health in TBI survivors. However, a systematic investigation into the long-term development of somatic comorbidities has not yet been undertaken.

This study aimed to investigate the burden of somatic health in adults who have survived a TBI by utilizing a nationwide cohort spanning 25 years. Using age-matched non-TBI controls as reference for comparison (“Controls”), the primary objectives were to (1) analyze the association between experiencing a TBI and the long-term risk of selected somatic comorbidities defined within the Charlson Comorbidity Index (CCI), and to (2) investigate whether the mean cumulative CCI score is higher in TBI survivors compared to Controls post-index, specifically accounting for the disease severity by using the corresponding weight within the CCI framework.

## Methods

### Study population

The study population has been described in more details elsewhere [35]. In brief, we included patients that experienced and survived a TBI between January 1 st, 1994 and the administrative end date December 31 st, 2018. Surviving a TBI was defined as surviving beyond discharge from the hospital stay specifically associated with the TBI. This included transfers between hospital units during the hospitalization (e.g., emergency unit, neurosurgical department, neuro-intensive care unit, and neurological department), but excluded any post-discharge rehabilitation stays. Patients were identified in the Danish National Patient Registry by using ICD-10 codes as listed in Supplementary Table 1 [29]. The Danish Patient Safety Authority approved the project (j. nr.: 3–3013-2784/1).

We excluded individuals who had any TBI recorded before January 1^st^, 1994 (using ICD-8 codes, Supplementary Table 1). TBI patients who were involved in a multiorgan trauma were excluded from the study population since accumulation of comorbidities could result from other concurrent injuries rather than the TBI itself. Multiorgan trauma was defined by the ICD-10 codes T02.x to T14.x and when recorded on the TBI index date.

### Matching of TBI survivors and Controls

Each TBI survivor was age-matched (± 3 days) with five TBI-naïve Controls from the general population. Data on TBI survivors and Controls were retrieved from Danish national registries using data linkage through unique civil security numbers.

### Age groups

TBI cases and Controls were categorized into age groups to better account for natural differences across life stages, such as age-related accumulation of comorbidities. The age groups were as follows: the youngest (18.0–39.9 years); the middle (40.0–69.9 years); and, the oldest (≥ 70.0 years).

### TBI severity groups and TBIs combined

In our analysis, we differentiate between “*TBI severity groups*” and “*TBIs combined*”.

1. *TBI severity groups* were categorized based on length of stay (LOS) to enable a more nuanced analysis by distinguishing between less and more severe TBIs. While measures such as the Glasgow Coma Scale would have been ideal, these are not available in Danish national registries. However, it is generally not recommended to use LOS as a proxy for TBI severity in register-based studies, as LOS is influenced by multiple factors beyond injury severity (particularly in elderlies) [35]. Therefore, LOS-based TBI severity classification was used exclusively for explorative purposes. Still, we added this approach because shorter hospital stays may reflect milder injuries or brief observation, whereas longer stays may indicate more severe injuries in a broader clinical context [35]. In this context, we considered three *TBI severity groups*: 1 day (1d TBIs), 2–3 days (2–3d TBIs), and ≥ 4 days of LOS (≥ 4 d TBIs), which we have described in detail previously [35].
2. *TBIs combined* includes all TBI survivors as a single group, regardless of their individual LOS.

### Outcomes

First, we selected four comorbidities for further investigation. Cerebrovascular disease was selected due to its direct link to the brain. Cancer, diabetes, and cardiovascular were selected for their relevance to the public health burden. Dementia will not be covered herein since it has already been explored using Danish register-based data [11]. Secondly, we included *all* conditions described within the CCI framework, and used their corresponding weight (CCI-score; Supplementary Table 2) as a metric to quantify the burden of somatic comorbidities accumulated over time [6, 39, 45].

### Statistics

Time since index was chosen as the underlying time scale, and all analyses were stratified for each age group. All analyses were performed using the statistical software* R* v. 4.3.

### The risk of selected comorbidities

Each of the four selected comorbidities was analyzed separately. TBI survivors and Controls who had experienced the event *prior* to the index date were excluded from analysis of that specific event. Individuals who experienced the event within 30 days of index were censored at that time to mitigate the risk of pre-existing conditions being coincidentally diagnosed during the hospital stay [16]. For cerebrovascular events, censoring patients with such an event within 30 days of index was essential to prevent modification of initial diagnoses from being misinterpreted as new events in this register-based design.

The Aalen-Johansen method, considering event-free death as a competing risk, was used to estimate the unadjusted cumulative proportion of the event [2, 3, 14, 34, 38]. To adjust for confounders, we obtained odds ratios by using a multiple binomial regression model (comparing both *TBI severity groups* and *TBIs combined* to Controls). The model was fitted using inverse probability of censoring weighting with the censoring weights computed using the Kaplan–Meier method, further stratified on a categorical variable with five levels reflecting the 5-year interval in which the index date occurred: (level 1) 1994–1998, …, (level 5) 2014–2018 [4, 41]. The model was adjusted for: baseline comorbidity (CCI-score sum: 0, 1, or ≥ 2), age (continuously), sex, education level (unknown, compulsory, vocational, 5–7.5 years, or ≥ 8 years beyond compulsory), demographic region (capital, Zealand, North Jutland, Central Jutland, South Denmark), ethnicity (Danish, Western, or non-Western), cohabitant status (number of adults ≥ 25.0 years living on the same address as the TBI survivor: 0 or ≥ 1), and, for the 18.0–39.9 group, parental education (categories from unknown to both having ≥ 8 years completed post-compulsory).

### The cumulative burden of somatic comorbidity

The endpoint was accumulation of CCI-score(s) over time as a proxy for the total burden of somatic comorbidities developed since index. Hence, *all* conditions within the CCI framework, and their corresponding CCI-score weights, were included in this analysis (Supplementary Table 2). A comorbidity was only included once and at the time of its first occurrence post-index. Thus, the development of a CCI condition was only considered a new development if it had not been documented prior to the index date. We considered the development of any new somatic comorbidity as a “recurring event”, reflecting the possibility that an individual may develop as many new comorbidities as there are unique ICD-10 codes within the individual CCI conditions—but only once, only post-index and only the main ICD-10 code category (e.g., E10 as one unique ICD-10 code, not each of E10.1, E10.2, …). Death was treated as a terminal event, where individuals were censored alive either at the administrative end date, or at the date of emigration (if emigrated). Thus, we used the Ghosh-Lin marginal regression model for recurrent and terminal events [15]. Furthermore, the weighted CCI-score allows for more serious conditions to have greater influence on the cumulative burden of somatic conditions, which was used in Ghosh-Lin model to weight each recurrent event (i.e., the burden of any new CCI condition was weighted according to its CCI score [Supplementary Table 2]) [32].

We adjusted the model as described for the binomial regression, thereby ensuring that the baseline sum of CCI-score(s) was accounted for at index. Hence, the weighted Ghosh-Lin model estimates the mean cumulative CCI-score over time given these adjusted risk covariates. Finally, the reported outcome is the ratio of mean cumulative CCI-scores for each individual *TBI severity group* with Controls as reference.

## Results

The cohort included 905,401 individuals, of whom 153,177 experienced and survived a TBI (characteristics in Table 1). The total follow-up time was 9,909,012 person-years, with a median follow-up of 13.3 years [42].

**Table 1:** Summary of the covariate distribution for age-matched non-TBI Controls and the TBI cohort

Categories	Control *n* = 752,224	TBI *n* = 153,177
Total person-years followed (median follow-up*, 95% CI)	8,296,876 (13.3 yrs, 13.2–13.3)	1,612,137 (13.4 yrs, 13.4–13.5)
Age (median, interquartile range**)	43.4 (26.6, 63.0)	43.5 (26.5, 63.6)
Sex		
Female	385,627 (51.3%)	69,639 (45.5%)
Male	366,597 (48.7%)	83,538 (54.5%)
Sum of CCI-score at index		
0	627,293 (83.4%)	116,900 (76.3%)
1	69,113 (9.2%)	20,543 (13.4%)
> 2	55,818 (7.4%)	15,734 (10.3%)
Educational attainment		
Unknown	106,752 (14.2%)	18,195 (11.9%)
Compulsory	250,056 (33.2%)	56,739 (37.0%)
Vocational	274,764 (36.5%)	54,488 (35.6%)
Compulsory + 5.0 to 7.5 years	93,763 (12.5%)	17,866 (11.7%)
Compulsory + ≥ 8.0 years	26,889 (3.6%)	5,889 (3.8%)
Residential region		
Capital	143,015 (19.0%)	48,467 (31.6%)
Region Zealand	149,738 (19.9%)	23,631 (15.4%)
Central Jutland	167,890 (22.3%)	33,471 (21.9%)
North Jutland	104,408 (13.9%)	16,383 (10.7%)
South Denmark	187,173 (24.9%)	31,225 (20.4%)
Ethnicity		
Danish	674,520 (89.7%)	140,366 (91.6%)
Western	40,856 (5.4%)	5,933 (3.9%)
Non-western	36,848 (4.9%)	6,878 (4.5%)
Adults living on address***		
0	618,209 (82.2%)	115,547 (75.4%)
> 1	134,015 (17.8%)	37,630 (24.6%)
Year group of index date		
1994 to 1998	168,630 (22.4%)	33,866 (22.1%)
1999 to 2003	160,947 (21.4%)	32,481 (21.2%)
2004 to 2008	143,361 (19.1%)	29,250 (19.1%)
2008 to 2013	132,939 (17.7%)	27,455 (17.9%)
2014 to 2018	146,347 (19.5%)	30,125 (19.7%)

^*^estimated using reverse Kaplan–Meier. ** interquartile range is between the 25th and 75th quantile

^***^ adults ≥ 25.0 years in addition to the TBI patient

95% CI (95% confidence interval). Yrs (years). CCI (Charlson Comorbidity Index at TBI index)

### Distribution of CCI conditions

**Fig. 1:**
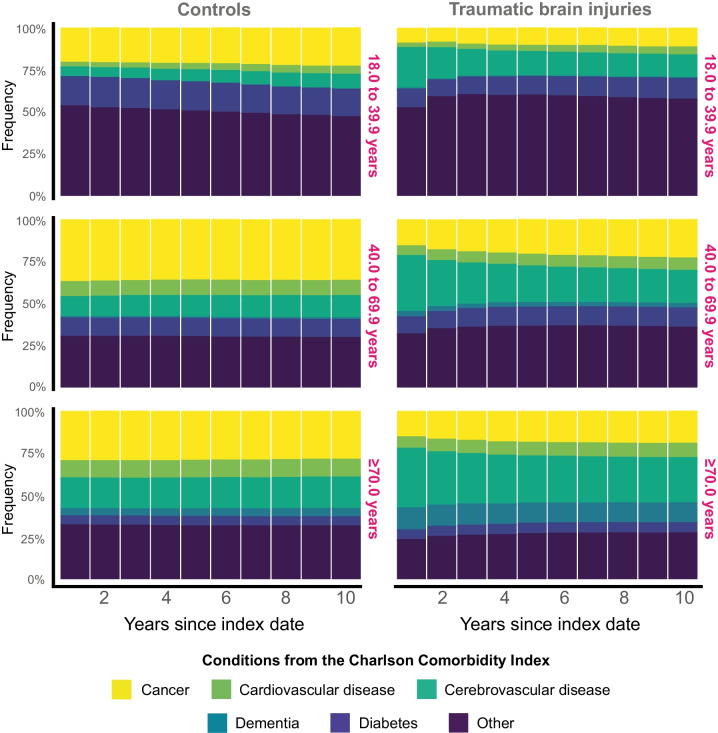
The crude distribution of comorbidities shown in successive 1-year intervals over the 10-year post-index period. The comorbidities included only those recorded after index date, imposing that its first documentation was registered post-index. Conditions that pre-dated the index date were excluded and were not eligible for post-index registration for that specific individual

Figure 1 shows that the crude distribution of new post-index comorbidities remained relatively stable over time in Controls, with cancer being the most prevalent condition. TBI survivors, however, experience a significant increase in cerebrovascular disease, emerging as the primary comorbidity throughout the initial 10-year timeframe.

### Risk of selected comorbidities

#### Cerebrovascular disease

The risk of cerebrovascular disease was significantly increased and showed a strong correlation with the *TBI severity groups*, irrespective of the age groups (Supplementary Fig. 1). Therefore, we conducted a more detailed post-hoc analysis focusing on non-traumatic intracerebral hemorrhage (*ICH.* ICD-10: I61.x and I62.x) and cerebral infarction (ICD-10: I63.x).

#### ICH

##### TBI severity groups

**Fig. 2:**
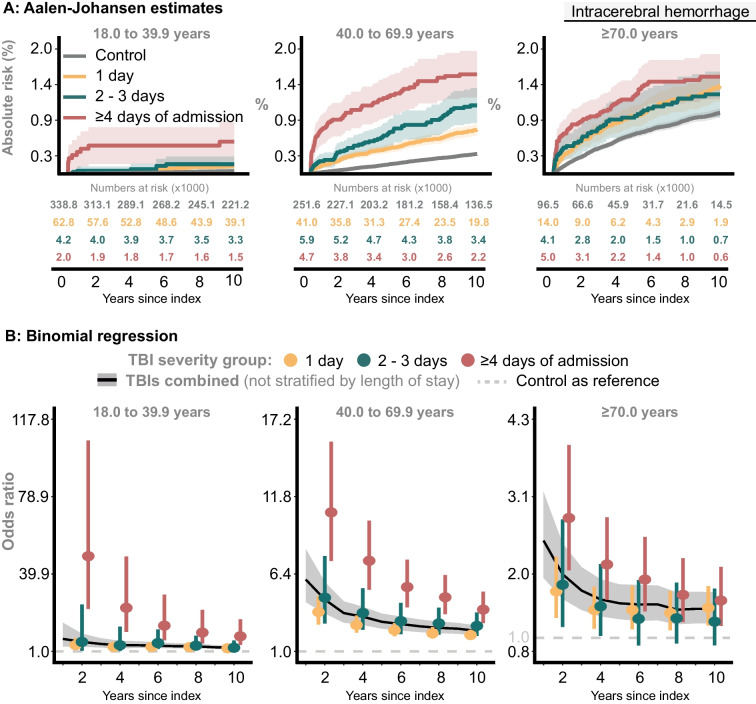
The risk of non-traumatic intracerebral hemorrhage (ICD10: I61, I62).** A**: Proportion of Controls and individuals in the *TBI severity groups* with intracerebral hemorrhage. **B**: The probability of intracerebral hemorrhage at specific time points, comparing the *TBI severity groups* with Controls

In the youngest age group, the proportion of ICH was higher among ≥ 4 d TBIs than any of: 1 d TBIs, 2-3d TBIs, and Controls, which had similar estimates. For the middle and oldest age group, there was a higher proportion of ICH occurring in TBI survivors than in Controls (Fig. 2A). In all age groups, the adjusted odds ratios for ≥ 4 d TBIs remained significantly higher compared to Controls and any remaining *TBI severity group* (Fig. 2B).When comparing ≥ 4 d TBIs with Controls, the 10-year adjusted odds ratio remained high at 8.59 (95% CI: 4.30 to 17.18, *P* < 0.0001) for the youngest, 3.92 (95% CI: 2.97 to 5.16, *P* < 0.0001) for the middle, and 1.57 (95% CI: 1.18 to 2.08, *P* = 0.002) for the oldest age group.

##### TBIs combined

The 10-year adjusted odds ratios were 2.84 (95% CI: 2.09 to 3.84, *P* < 0.0001), 2.43 (95% CI: 2.11 to 2.80, *P* < 0.0001), and 1.44 (95% CI: 1.23 to 1.70, *P* < 0.0001), for the youngest, middle, and oldest age group (Fig. 2B).

#### Exploratory post-hoc analysis: experiencing multiple TBIs over time and ICH

Given the consistent association observed between TBI severity and ICH across age groups, we conducted an exploratory post-hoc analysis to evaluate whether experiencing multiple TBIs over time was associated with an increased rate of ICH. This was not a pre-specified aim, thus explorative entirely and should be interpreted as hypothesis-generating.

We applied a time-dependent cause-specific Cox regression model, in which the number of TBIs was allowed to accumulate over time as a time-varying covariate (Table 2). Hence, we categorized individuals by the number of TBIs since index with 0 as *Controls*, 1 (index TBI), 2, or ≥ 3 TBIs sustained. To reduce misclassification of ICH events related to initial trauma, ICH occurring within 30 days of any TBI was treated as a competing event. Death without prior ICH was also treated as a competing event. Models were adjusted to confounders as in the primary analyses.

**Table 2:** Post-hoc exploratory analysis

Number of TBIs	Hazard rate ratios (95% confidence interval, *P*-value)		
	18.0 to 39.9 years	40.0 to 69.9 years	≥ 70.0 years
1 TBI (index TBI) Reference *Controls* (0 TBI)	1.69 (1.34 to 2.12, *P* < 0.0001)	2.03 (1.85 to 2.24, *P* < 0.0001)	1.48 (1.33 to 1.65, *P* < 0.0001)
2 TBIs Reference *Controls* (0 TBI)	3.93 (2.19 to 2.24, *P* < 0.0001)	3.71 (2.85 to 4.83, *P* < 0.0001)	2.17 (1.54 to 3.06, *P* < 0.0001)
≥ 3 TBIs Reference *Controls* (0 TBI)	11.82 (5.74 to 24.38, *P* < 0.0001)	6.91 (4.56 to 10.48, *P* < 0.0001)	4.19 (2.26 to 7.76, *P* < 0.0001)

ICH: non-traumatic intracerebral hemorrhage, TBI: traumatic brain injury

We examined the association between cumulative TBIs and the rate of non-traumatic intracerebral hemorrhage (ICH). The number of TBIs since index was counted and modeled categorically (0 = *Controls*; 1 (index TBI), 2, or ≥ 3 TBIs). TBI was defined as any of the ICD-10 codes listed in Supplementary Table 1. We applied a time-dependent cause-specific Cox regression model to estimate hazard rate ratios. To minimize misclassification of early ICH events potentially related to initial trauma, ICH occurring within 30 days of any TBI was treated as a competing event. Death without prior ICH was also handled as a competing event. The model was adjusted using the same covariates as described in the Methods for the binomial and recurrent event regressions (covariate coefficients are not shown)

We found that the rate of ICH increased gradually and significantly with the number of TBIs experienced. Compared to *Controls*, the hazard rate ratios for those with ≥ 3 TBIs were markedly elevated across all age groups: 11.82 (95% CI: 5.74 to 24.38, *P* < 0.0001) in the youngest, 6.91 (95% CI: 4.56 to 10.48,* P* < 0.0001) in the middle, and 4.19 (95% CI: 2.26 to 7.76, *P* < 0.0001) in the oldest group (Table 2). These findings suggest a strong dose–response pattern between cumulative TBI exposure and developing ICH, warranting further investigation in future studies.

#### Cerebral infarction

##### TBI severity groups

**Fig. 3:**
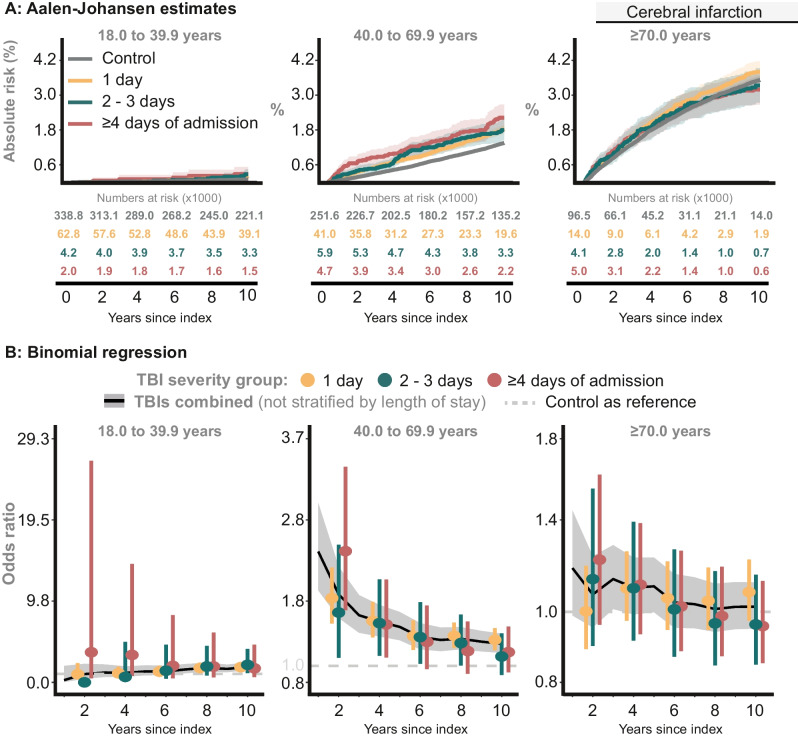
The risk of cerebral infarction (ICD10: I63)**. A**: Proportion of Controls and individuals in the *TBI severity groups* with cerebral infarction. **B**: The probability of cerebral infarction at specific time points, comparing the *TBI severity groups* with Controls

The proportion of cerebral infarction was low in the youngest age group, regardless of TBI patient or Control. In middle age group, the proportion of TBI survivors with cerebral infarction was higher than Controls; however, no clear differentiation between the individual *TBI severity groups* was observed. In contrast, among the oldest age group, the proportion of individuals with cerebral infarction was comparable between TBI survivors and Controls (Fig. 3A). These observations remained consistent in the adjusted analyses (Fig. 3B).

##### TBIs combined

The 10-year adjusted odds ratios were as follows: 1.84 (95% CI: 1.44 to 2.36, *P* < 0.0001), 1.27 (95% CI: 1.16 to 1.39,* P* < 0.0001), and 1.02 (95% CI: 0.92 to 1.13, *P* = 0.66) for the youngest, middle, and oldest age group.

#### All-type cancer (with and without metastases)

##### TBI severity groups

**Fig. 4:**
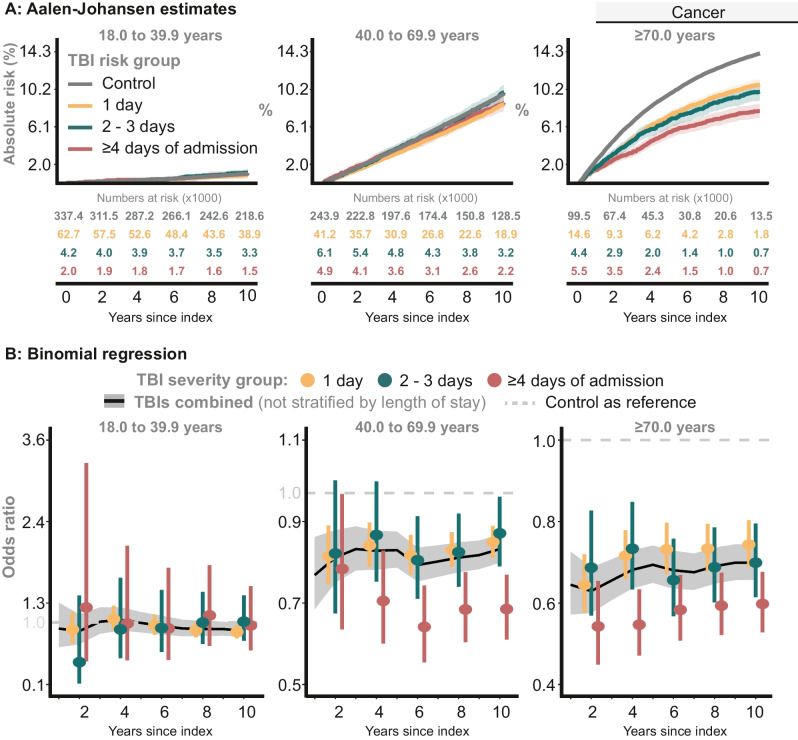
The risk all-type cancer (with and without metastasis) as defined within the CCI framework (Supplementary Table 2). **A**: Proportion of Controls and individuals in the *TBI severity groups* with cancer. **B**: The probability of cancer at specific time points, comparing the *TBI severity groups* with Controls

The proportion of individuals with cancer was notably lower in TBI survivors than Controls, and was particularly pronounced for TBI survivors older than 70.0 years (Fig. 4A). For patients in the middle and oldest age groups, ≥ 4 d TBIs had favorable adjusted odds ratios that reflect a reduced risk of cancer compared with Controls (Fig. 4B).

##### TBIs combined

For the middle and oldest age groups, the 10-year adjusted odds ratios were 0.86 (95% CI: 0.82 to 0.89, *P* < 0.0001) and 0.71 (95% CI: 0.67 to 0.75, *P* < 0.0001), respectively. No difference was observed for the youngest age group (Fig. 4B).

### Post-hoc analysis: the lower risk of cancer

As this finding was surprising, we undertook a *post-hoc* analysis (not shown). A reduction in cancer risk is not immediately apparent as being biologically correlated with TBIs and is suspicious of TBI patients being underdiagnosed. In general, the risk of cancer was particularly lower regarding pathologies that frequently relies on routine screening programs, e.g., a significantly lower proportion of females with ≥ 4 d TBIs have breast cancer 5-years post-index (in the middle age group), while no significant differences were detected between Controls, 1 d and 2-3d TBIs. It has been documented previously that accumulated morbidity negatively impact breast cancer screening, supporting this observation [33, 46].

#### Diabetes

##### TBI severity groups

In the youngest and oldest age groups, the proportion of individuals developing diabetes were similar across the *TBI severity groups* and Controls. In contrast, the proportion of individuals with diabetes in the middle age group was significantly higher across the TBI survivors when compared to Controls (Supplementary Fig. 2A).

##### TBIs combined

In the youngest and middle age groups, the adjusted analysis showed that TBI survivors have a higher long-term risk of developing diabetes. The 10-year adjusted odds ratios for developing diabetes were 1.14 (95% CI: 1.01 to 1.28, *P* = 0.03), 1.24 (95% CI: 1.16 to 1.33, *P* < 0.0001) and 0.98 (95% CI: 0.87 to 1.11, *P* = 0.76), for each corresponding age group (Supplementary Fig. 2B).

#### Cardiovascular disease

##### TBI severity groups

The proportion of cardiovascular disease was higher among TBI survivors than Controls for the youngest population. For the oldest population, TBI survivors may have slightly better outcomes compared to Controls (Supplementary Fig. 3A). There was no association between cardiovascular disease and the *TBI severity groups* in the adjusted analysis.

##### TBIs combined

Furthermore, in the adjusted analysis, the 10-years odds ratios were as follows for the youngest: 1.51 (95% CI: 1.29 to 1.76, *P* < 0.0001), the middle: 0.97 (95% CI: 0.93 to 1.07, *P* = 0.46) and the oldest age group: 0.91 (95% CI: 0.84 to 0.98, *P* = 0.01) (Supplementary Fig. 3B).

## All CCI-conditions combined: the mean cumulative CCI-score

Table 3 presents mean cumulative CCI score ratios derived from a recurrent event regression model with death treated as a terminal event. Estimates reflect the relative burden of newly developed somatic comorbidities across covariate strata, adjusted for all other variables in the model.

**Table 3:**
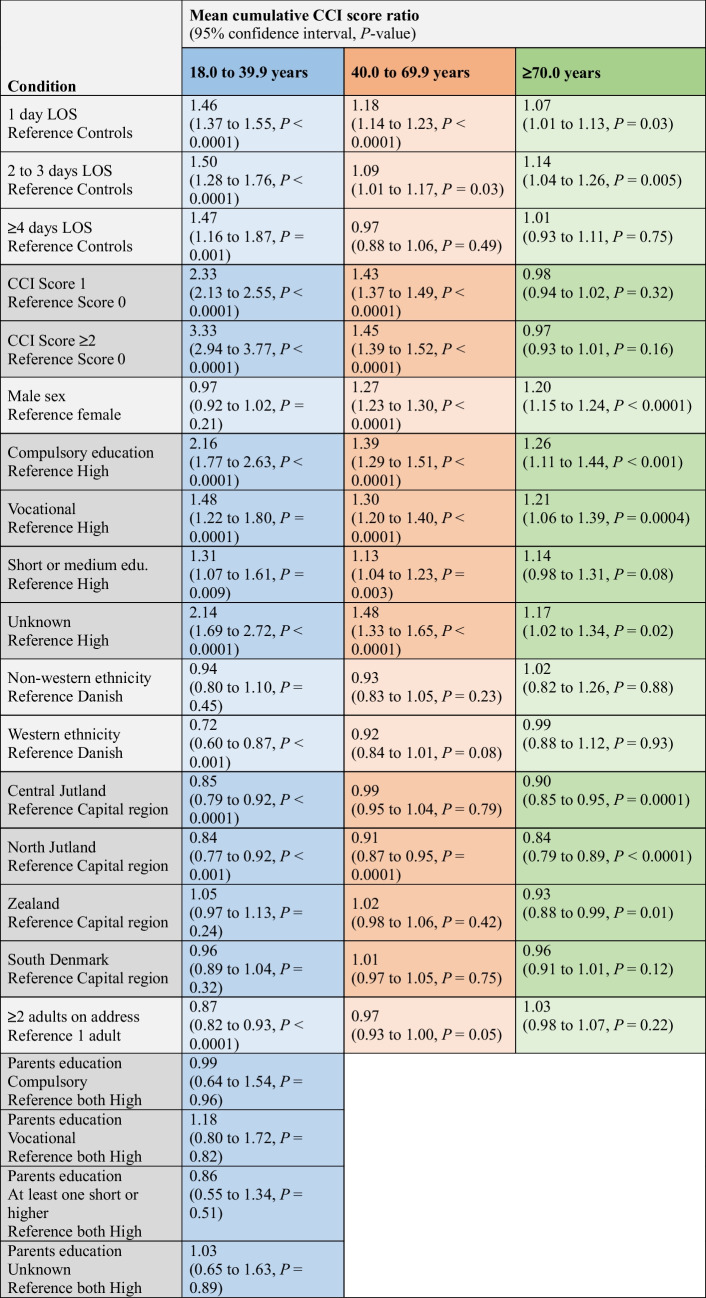
Recurrent event regression with death as terminal event. Mean cumulative CCI score ratios for each covariate were obtained across age groups. Conditions diagnosed within 30 days from index were treated as a competing event to avoid surveillance bias inflating the mean cumulative CCI-score for admitted TBI patients

For the youngest age group, 1 d TBIs, the mean cumulative CCI-score increased by 46% (95% CI: 1.37 to 1.55, *P* < 0.0001), by 50% for 2-3d TBIs (95% CI: 1.28 to 1.76, *P* < 0.0001), and by 47% for ≥ 4 d TBIs (95% CI: 1.16 to 1.87, *P* = 0.001) (Table 3).

In the middle-aged group, both 1 d and 2–3d TBIs were associated with significantly higher mean cumulative CCI scores, increasing by 18% (95% CI: 1.14 to 1.23, *P* < 0.0001) and 9% (95% CI: 1.01 to 1.17, *P* = 0.03). The ≥ 4 d TBIs was not associated with a significant difference.

For the oldest age group, the mean cumulative CCI scores was significantly higher in both 1 d and 2–3d TBI (1.07, 95% CI: 1.01 to 1.13, *P* = 0.03; and, 1.14, 95% CI: 1.04 to 1.26, *P* = 0.005, respectively), but not ≥ 4 d TBIs (Table 3).

### Post-hoc sensitivity analysis: with and without cancer diagnoses

The possible underdiagnosis of cancer risk in TBI survivors may bias the mean cumulative CCI score. Therefore, we conducted a *post-hoc* sensitivity analysis excluding cancer diagnoses from the CCI framework (Table 4; which presents mean cumulative CCI score ratios similarly to Table 3, but where cancer with and without metastases has been removed from the model). Except for ≥ 4 d TBIs in the oldest group, all *TBI severity groups* had significantly higher mean cumulative CCI scores than Controls, which was substantially more pronounced than in the main analysis.

**Table 4:** Sensitivity analysis. Recurrent event regression with death as terminal event, but where cancer diagnoses – with or without metastasis – are excluded. Otherwise, performed and adjusted as shown in Table 4; however, only TBI risk group estimates are shown

Condition	Mean cumulative CCI score ratio (95% confidence interval, *P*-value)		
	18.0 to 39.9 years	40.0 to 69.9 years	≥ 70.0 years
1 day LOS Reference Controls	1.64 (1.53 to 1.75, *P* < 0.0001)	1.30 (1.25 to 1.36, *P* < 0.0001)	1.09 (1.03 to 1.16, *P* = 0.007)
2 to 3 days LOS Reference Controls	1.85 (1.54 to 2.22, *P* < 0.0001)	1.28 (1.17 to 1.40, *P* < 0.0001)	1.15 (1.04 to 1.28, *P* = 0.008)
≥ 4 days LOS Reference Controls	1.44 (1.09 to 1.89, *P* = 0.009)	1.20 (1.08 to 1.33, *P* < 0.001)	1.05 (0.95 to 1.16, *P* = 0.32)

## Discussion

In this study, we focused on TBI survivors without concurrent extracranial injuries at index. Three novel key findings were (1) the shift in distribution of somatic comorbidities occurring after index, with cerebrovascular disease demonstrating a considerable long-term impact on TBI survivors, (2) cancer appears to be underdiagnosed in TBI survivors, implying that a substantial burden of somatic comorbidity may remain unrecognized; (3) still, and across all age groups, TBI survivors accumulate a significantly higher burden of somatic comorbidities post-index, as reflected by the higher mean cumulative CCI score—regardless of whether the CCI-score weight of cancer is taken into account.

Our findings suggest that health-related challenges in TBI survivors extend far beyond the immediate TBI-related disabilities. The accumulation of somatic comorbidities is an indirect consequence of TBIs that remain insufficiently recognized for its potential role in deteriorating somatic health in survivors, further exacerbating YLD.

LOS was used exploratively as a proxy to reflect that more severe TBI cases likely result in prolonged hospitalizations (not including rehabilitation). Though this is not equivalent to standardized clinical severity scoring, adjustment for confounders such as comorbidity, sex, and educational level was applied to account for factors influencing LOS. Thus, in this context, LOS-based TBI severity groups may still descriptively provide some insight into the possible association between TBI severity and the risk of developing somatic comorbidities.

## Cerebrovascular disease related to TBIs

Post-hoc analysis (not shown) indicated that skull fractures were linked to a higher risk of cerebral infarction, but did not consistently correspond with longer LOS. This may explain why TBIs show an increased risk of cerebral infarction without associating with severity.

The association between *TBI severity groups* and an increased risk of ICH is possibly related to a range of biological processes. Local TBI-induced inflammation, combined with vascular damage, may weaken cerebral vessels or lead to the formation of new, more fragile vessels, consequently making them more prone to rupture. In this context, observational studies have reported that hypertension is one of the most common post-TBI conditions [9, 27]. This is important because hypertension can further stress fragile vessels, synergistically increasing the risk of ICH.

Previous studies have reported an increased risk of stroke following a TBI, although limited in cohort size and/or follow-up time. A case–control study (23,199:69,597) reported that the 5-year post-index risk of all-type stroke was 2.3 times higher in TBI survivors [1]. With 13 years follow-up, another case–control study (16,211:32,422) reported hazard ratios of 6.0 for ICH and 2.1 for cerebral infarction [7]. Finally, a study compared 436,630 TBI cases to ~ 736,723 non-TBI trauma cases and found a hazard ratio of 1.3 for cerebral infarction [5].

## Surveillance bias and risk of developing comorbidity

Surveillance bias was mitigated by treating comorbidities recorded within 30 days post-index as “competing events”, consequently causing an *immortal time bias*-like effect where new comorbidities cannot be diagnosed within the first month – which is acknowledged as a minor but necessary limitation.

Still, TBI survivors may receive prolonged medical attention after discharge—likely exceeding that of the general population. Hence, the recording of somatic comorbidities post-index may still be attributed to more intensive surveillance. While this could affect diagnosis of diseases that rely on paraclinical methods, such as diabetes, ICH and cerebral infarction are characterized by distinct symptomatology involving paralysis, severe headache, vomiting, loss of consciousness, seizures, or aphasia. Because of these visible symptoms, we consider surveillance bias to have a minimal impact on the diagnosis of these conditions. Finally, multiple factors in fact support a causal link between TBI and ICH, including consistency with existing literature (though scarce), temporality, a dose–response relationship (the gradual correlation with *TBI severity groups*), and biological plausibility (hypertension combined with TBI-induced vascular fragility). Furthermore, the exploratory post hoc analysis suggested a dose-dependent increase in the hazard rate of ICH with increasing number of TBIs. Given that this analysis was not prespecified and exploratory in nature, the findings should be interpreted as hypothesis-generating.

In addition, we acknowledge that comorbidity burden, as captured by ICD codes, may partly reflect healthcare utilization patterns, which vary by socioeconomic position [12, 13, 28]. Individuals with frequent healthcare contact are more likely to receive diagnostic codes whereas those with limited utilization may have undiagnosed illnesses. This may lead to differential misclassification of comorbidity burden. Although we adjusted for key socioeconomic proxies, including educational attainment, geographic region, and summed baseline CCI-score, residual bias due to differences in diagnostic coding or detection remains possible.

## TBIs as a mechanism of health inequality

We consider that TBIs are unrecognized for their mechanisms in driving health inequality: the (1) early functional decline and (2) reduced healthcare utilization [18]. First, the higher burden of somatic comorbidities accelerates early functional decline in TBI survivors, here referring to a premature reduction in physical and functional abilities relative to natural aging. Secondly, underdiagnosis of cancer may occur due to reduced healthcare utilization, for example, when cognitively impaired patients face challenges in communicating symptoms or fail to participate in routine screening programs—thereby leading to delayed or missed cancer detection. In this context, experiencing a TBI may drive health inequalities compared to Controls. This could be supported by previous studies suggesting that higher morbidity is associated with lower screening participation in breast cancer patients [33, 46]. As such, cancer may be underdiagnosed in many TBI survivors, potentially leading to a substantial underestimation of their cumulative burden of somatic comorbidity. However, addressing this issue may be particularly challenging in patients unable to provide informed consent, as coercing cognitively impaired individuals to diagnostic procedures would raise ethical concerns and potentially violate their rights. This could, in part, contribute to the observed lower cancer risk in TBI patients (particularly those with more severe injuries); however, this remains a hypothesis and should be interpreted as a plausible but unproven explanation for the observed cancer patterns in this population.

## ICD-10 codes as metric for somatic comorbidity burden

While the CCI framework remains one of the most widely used comorbidity metrics in registry-based epidemiological research, we acknowledge its limitations [6, 39]. Some of the CCI conditions—such as AIDS—may no longer carry the same mortality risk as when the index was developed, and it does not capture frailty or functional status [37]. Alternative tools such as the Elixhauser Comorbidity Index include a broader range of comorbidities and have shown superior predictive performance in several hospital-based studies, particularly for in-hospital mortality and complications [44, 47]. Frailty-based indices, including the Hospital Frailty Risk Score (HFRS) and the modified Frailty Index (mFI), have demonstrated enhanced prognostic value for adverse outcomes, especially in surgical and older populations [20, 36]. Clinician-assessed metrics like the American Society of Anesthesiologists (ASA) score incorporate both comorbidities and functional status settings [25]. Specifically within TBI, recent evidence shows the ASA score is independently associated with 90-day mortality after complicated mild and moderate-to-severe TBI [25, 26]. However, the ASA score is not systematically available in Danish national registries and is associated with substantial missing values across geography and calendar time. Thus, several approaches to account for comorbidity, frailty, and functional status were considered when designing the present study. The CCI framework was prioritized because it is available and based on objectively recorded ICD codes, making it reproducible, scalable, and feasible for use in this nationwide register-based setting. Nonetheless, we acknowledge the limitations from using ICD-10 codes.

## Strength and limitation

The nationwide cohort design, with a 1:5 ratio of Controls, allowed for longitudinal tracking of both TBI survivors and Controls, facilitating robust comparisons with adjustments to temporal changes. Two key strengths of this study were the (1) the exclusion of multiorgan trauma patients and (2) incorporating a proxy of TBI severity, enabling a more detailed analysis of TBI-related development of somatic comorbidity.

Although expanding the study to encompass the entire national population as a full-scale nation cohort might have allowed for slightly more detailed analyses, maintaining the 1:5 age-matching poses only a minimal limitation. Finally, the lack of detailed clinical data on actual TBI severity remains a key limitation. Specifically, registry-based epidemiological studies carry inherent risks of misclassification when based on ICD-10 codes—this is a universal limitation. However, a validation study of ICD-10-CM surveillance used codes for hospitalized TBI patients found that the most frequently used codes—such as S02 and S06—had positive predictive values ranging from 82.1% to 92.5% [48]. This indicates that, depending on the specific ICD-10 code, more than 8 to over 9 out of 10 coded cases reflect true clinical TBI diagnoses. This suggests a high level of coding accuracy for hospitalized TBI patients in this specific setting, meaning the presence of such a code in registry data corresponds to a true clinical diagnosis. Although limited to a single study, these findings may provide reassurance that, despite the inherent limitations of administrative data, ICD-10-based definition of TBIs are generally robust in capturing true cases. Nonetheless, we acknowledge that some degree of misclassification remains possible.

Finally, we excluded TBI patients with concurrent extracranial injuries to prioritize isolating the long-term somatic burden attributable specifically to the brain injury. While this exclusion mitigates confounding from other serious trauma-related conditions, it may also reduce the representation of high-energy trauma cases, such as those resulting from traffic accidents. Thus, although this methodological choice was necessary to support the primary aim of attributing comorbidity development to TBI itself, it may limit the generalizability of our findings to broader polytrauma populations.

## Conclusion

TBI survivors (without concurrent extracranial injuries) experience a higher burden of somatic comorbidities than the general population. This highlights how the indirect consequences of TBI extend far beyond the immediate post-index period, exacerbating YLD. TBIs emerge as an important risk factor for ICH. Recognizing these perspectives could have significant implications for individual and public health, given that many of these indirect consequences may be preventable through targeted interventions.

## Supplementary information

Below is the link to the electronic supplementary material.

ESM 1 (DOCX 3.26 MB)

ESM 2 (DOCX 3.26 MB)

ESM 3 (DOCX 3.26 MB)

ESM 4 (DOCX 3.26 MB)

ESM 5 (DOCX 3.26 MB)

## Data Availability

No datasets were generated or analysed during the current study.
