# Supplementary material for: The burden of somatic comorbidities in patients surviving a traumatic brain injury
Source: Acta Neurochir (Wien). 2025 Aug 7;167(1):216. doi: 10.1007/s00701-025-06617-1 (PMC12331845; doi:10.1007/s00701-025-06617-1)
Supplement: Supplementary file 1 — (DOCX 3.26 MB) [file 701_2025_6617_MOESM1_ESM.docx]

**Supplementary Figure 1.** *All-type* cerebrovascular disease as defined within the CCI framework (**Supplementary Table 2**). **A:** Proportion of Controls and individuals in the *TBI severity groups*. **B:** The probability of cerebrovascular disease at specific time points, comparing the *TBI severity groups* with Controls.

**
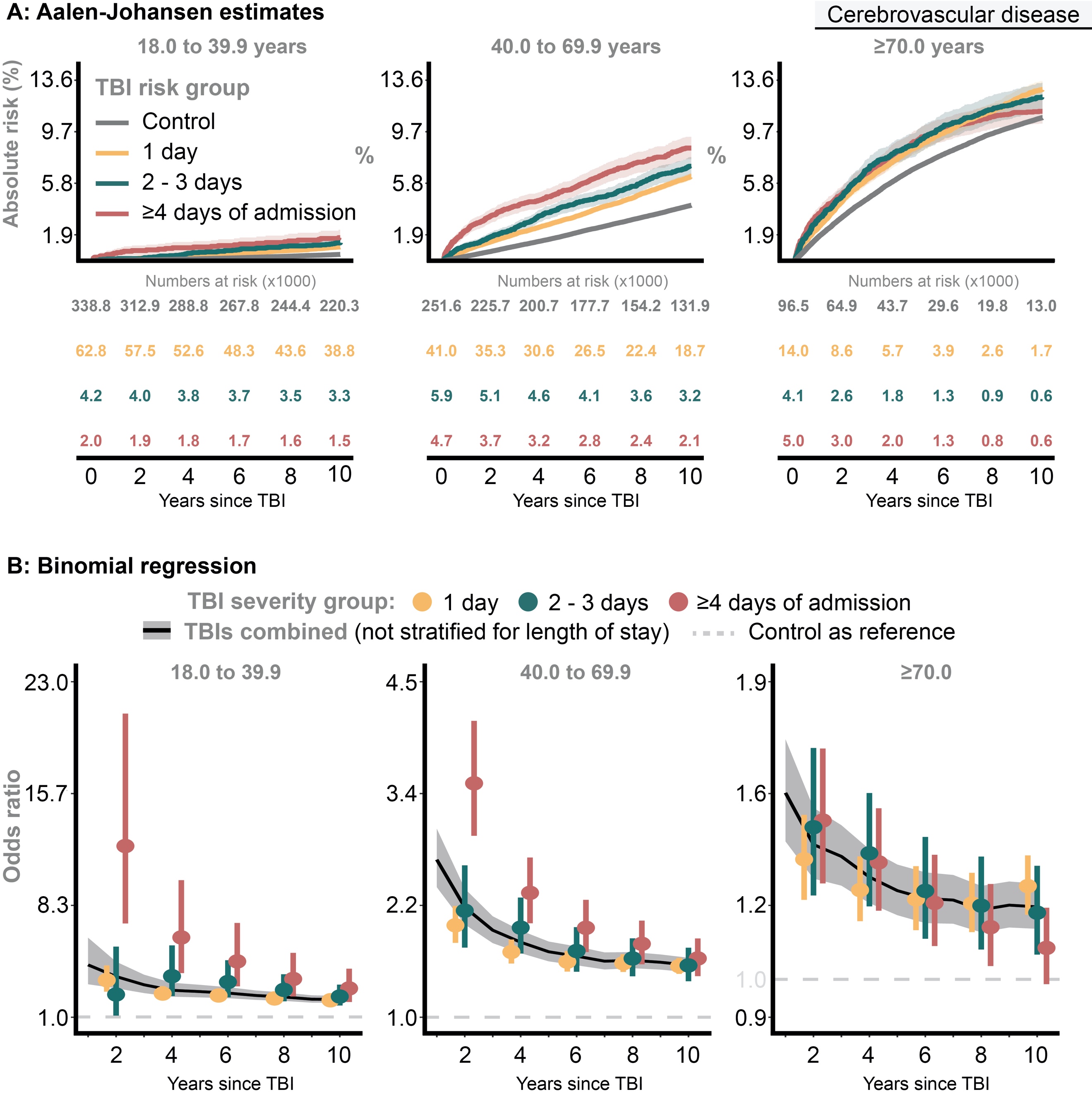
**
