# Supplementary material for: The burden of somatic comorbidities in patients surviving a traumatic brain injury
Source: Acta Neurochir (Wien). 2025 Aug 7;167(1):216. doi: 10.1007/s00701-025-06617-1 (PMC12331845; doi:10.1007/s00701-025-06617-1)
Supplement: Supplementary file 4 — (DOCX 3.26 MB) [file 701_2025_6617_MOESM4_ESM.docx]

| **Supplementary Table 1.** The ICD-8/-10 classification codes used to define a TBI before and after January, 1^st^, 1994. | |
| --- | --- |
| **The ICD-10 classification system**  Used between January, 1^st^, 1994 and December, 31^st^, 2018 | **Direct brain injuries**  ICD-10 code and description |
| S06.0 | Concussion |
| S06.1 | Traumatic cerebral oedema |
| S06.2 | Diffuse brain injury |
| S06.3 | Focal brain injury |
| S06.4 | Epidural hemorrhage |
| S06.5 | Traumatic subdural hemorrhage |
| S06.6 | Traumatic subarachnoid hemorrhage |
| S06.7 | Intracranial injury with prolonged coma |
| S06.8 | Other intracranial injuries |
| S06.9 | Intracranial injuries, unspecified |
| S07.1 | Crushing injury of skull |
|  | **Fracture(s) of skull and facial bones**  ICD-10 code and description |
| S02.0 | Fracture of vault of skull |
| S02.1 | Fracture of base of skull |
| S02.3 | Fracture of orbital floor |
| S02.7 | Multiple fractures involving skull and facial bones |
| S02.8 | Other fractures of skull and facial bones |
| S02.9 | Unspecified fracture of skull and facial bones |
| **The ICD-8 classification system**  Used before January, 1^st^, 1994 | **Direct brain injuries**  ICD-8 code and description |
| 85009 | Commotio cerebri,non compl. |
| 85099 | Commotio cerebri,sequelae |
| 85099 | Commotio cerebri |
| 85100 | Dilaceratio cerebri,non compl. |
| 85101 | Oedema cerebri traumaticum, non complicata |
| 85108 | Laesio traumatica cerebri alia definita,non complicata |
| 85109 | Contusio cerebri,non compl. |
| 85109 | Oedema cerebri traumaticum |
| 85110 | Dilaceratio cerebri,complicata |
| 85111 | Oedema cerebri traumaticum, complicata |
| 85118 | Laesio traumatica cerebri alia definita,complicata |
| 85119 | Contusio cerebri,complicata |
| 85119 | Contusio cerebri |
| 85129 | Dilaceratio cerebri |
| 85139 | Conquassatio cerebri |
| 85190 | Dilaceratio cerebri,sequelae |
| 85191 | Oedema cerebri traumaticum,sequelae |
| 85198 | Laesio traumatica cerebri alia definita,sequelae |
| 85199 | Contusio cerebri,sequelae |
| 85199 | Contusio, dilaceratio, conquassatio cerebri |
| 85200 | Haemorrhagia traumatica subarachnoidalis,non compl. |
| 85200 | Haemorrhagia traumatica subarachnoidalis |
| 85201 | Haemorrhagia(haematoma)traum. Subduralis,non complicata |
| 85201 | Haemorrhagia (haematoma) traumatica subduralis |
| 85202 | Haemorrhagia(haematoma)traum. Extraduralis,non complicata |
| 85202 | Haemorrhagia (haematoma) traumatica extraduralis |
| 85203 | Laesio traumatica vasis intracranialis |
| 85209 | Haemorrh.traum.subarachnoid., subdur.extraduralis,non compl. |
| 85209 | Haemorrh.traum.subarachnoid., subdur.,extraduralis |
| 85210 | Haemorrhagia traumatica subarachnoidalis,complicata |
| 85211 | Haemorrhagia(haematoma)traum. Subduralis,complicata |
| 85212 | Haemorrhagia(haematoma)traum. Extraduralis,complicata |
| 85219 | Haemorrh.traum.subarachnoid., subdur.extraduralis,complicata |
| 85290 | Haemorrhagia traumatica subarachnoidalis,sequelae |
| 85291 | Haemorrhagia(haematoma) traumatica subduralis,sequelae |
| 85292 | Haemorrhagia(haematoma) traumatica extraduralis,seq. |
| 85299 | Haemorrh.traum.subarachnoid., subdur.extraduralis,sequelae |
| 85309 | Haemorrh.traum.intracranialis alia s.non specif.,non compl. |
| 85319 | Haemorrh.traum.intracranialis alia s.non specif.,complicata |
| 85399 | Haemorrh.traum.intracranialis alia s.non specif.,sequelae |
| 85399 | Haemorrh.traum.intracranialis alia sive non specific. |
| 85402 | Haematotympanon,non complicata |
| 85409 | Laesio traumat.intracran.al.s. Non specif.non complicata |
| 85412 | Haematotympanon,complicata |
| 85419 | Laesio traumat.intracran.al.s. Non specif.complicata |
| 85490 | Arachnoiditis posttraumatica, sequelae |
| 85491 | Encephalopathia atrophica posttraumatica,sequelae |
| 85492 | Haematotympanon,sequelae |
| 85499 | Laesio traum.intracranialis alia sive non specif.sequelae |
| 85499 | Laesio traumatica intracranialis alia sive non specificata |
|  | **Fracture(s) of skull and facial bones**  ICD-8 code and description |
| 80009 | Fractura thecae cranii (frontal.parietalisinon compl. |
| 80019 | Fractura thecae cranii (frontal.parietalis)complicata |
| 80099 | Fractura thecae cranii (frontal.parietalis)sequelae |
| 80099 | Fractura cranii (frontalis,parietalis) |
| 80100 | Fractura ossis temporalis,non complicata |
| 80100 | Fractura ossis temporalis (pars squamosa excepta) |
| 80108 | Fractura baseos cranii alia definita,non complicata |
| 80108 | Fractura baseos cranii alia definita |
| 80109 | Fractura baseos cranii,non complicata |
| 80109 | Fractura baseos cranii |
| 80110 | Fractura ossis temporalis, complicata |
| 80118 | Fractura baseos cranii alia definita,complicata |
| 80119 | Fractura baseos cranii, complicata |
| 80190 | Fractura ossis temporalis, sequelae |
| 80198 | Fractura baseos cranii alia def.sequelae |
| 80199 | Fractura baseos cranii, sequelae |
| 80220 | Fractura orbitae |
| 80242 | Fractura orbitae, non compl. |
| 80300 | Fractura cranii c.haematomate extredur.traumatico,non compl. |
| 80301 | Fractura cranii c.haematomate subdurali traumat.non compl. |
| 80302 | Fractura cranii c.oedemate cerebri traumatico,non compl. |
| 80303 | Fractura cranii c.contusione cerebri,non complicata |
| 80304 | Fractura cranii c.contusione cerebri,non complicata |
| 80308 | Fractura cranii cum alia complicatione,non complicata |
| 80309 | Fractura cranii alia et non specificata,non complicata |
| 80310 | Fractura cranii c.haematomate extradur.traumatico,complicata |
| 80311 | Fractura cranii c.haematomate subdurali traumat.complicata |
| 80312 | Fractura cranii c.oedemate cerebri traumatico,complicata |
| 80313 | Fractura cranii c.contusione cerebri,complicata |
| 80314 | Fractura cranii c.contusione cerebri,complicata |
| 80318 | Fractura cranii cum alia complicatione,complicata |
| 80319 | Fractura cranii alia et non specificata,complicata |
| 80390 | Fract.cranii cum haematomate extredurali traumatico,seq. |
| 80391 | Fract.cranii cum haematomate subdurali traumatico,sequelae |
| 80392 | Fractura cranii cum oedemate cerebri traumatico,sequelae |
| 80393 | Fractura cranii cum contusione cerebri,sequelae |
| 80394 | Fractura cranii cum commotione cerebri,sequelae |
| 80398 | Fractura cranii cum alia complicatione,sequelae |
| 80399 | Fractura cranii alia et non specificata,sequelae |
| 80399 | Fractura cranii alia et non specificata |
| 80409 | Fractura cranii s.os.faciei c. Fract.os.aliorum,non compl. |
| 80419 | Fractura cranii s.os.faciei c. Fract.os.aliorum,complicata |
| 80499 | Fractura cranii s.os.faciei c. Fract.os.aliorum,sequelae |
