# Supplementary material for: The burden of somatic comorbidities in patients surviving a traumatic brain injury
Source: Acta Neurochir (Wien). 2025 Aug 7;167(1):216. doi: 10.1007/s00701-025-06617-1 (PMC12331845; doi:10.1007/s00701-025-06617-1)
Supplement: Supplementary file 5 — (DOCX 3.26 MB) [file 701_2025_6617_MOESM5_ESM.docx]

| **Supplementary Table 2.** Charlson Comorbidity Index. Included conditions, their respective weight and ICD-10 codes used for definition. | | |
| --- | --- | --- |
| **Condition** | **Weight (CCI score)** | **ICD-10 Codes** |
| Myocardial infarction | 1 | I21.x, I22.x, I25.2 |
| Congestive heart failure | 1 | I09.9, I11.0, I13.0, I13.2, I25.5, I42.0, I42.5-I42.9, I43.x, I50.x, P29.0 |
| Peripheral vascular disease | 1 | I70.x, I71.x, I73.1, I73.8, I73.9, I77.1, I79.0, I79.2, K55.1, K55.8, K55.9, Z95.8, Z95.9 |
| Cerebrovascular disease | 1 | G45.x, G46.x, H34.0, I60.x-I69.x |
| Dementia | 1 | F00.x-F03.x, F05.1, G30.x, G31.1 |
| Chronic pulmonary disease | 1 | I27.8, I27.9, J40.x-J47.x, J60.x-J67.x, J68.4, J70.1, J70.3 |
| Connective tissue disease | 1 | M05.x, M06.x, M31.5, M32.x-M34.x, M35.1, M35.3, M36.0 |
| Peptic ulcer disease | 1 | K25.x-K28.x |
| Mild liver disease | 1 | B18.x, K70.0-K70.3, K70.9, K71.3-K71.5, K71.7, K73.x, K74.x, K76.0, K76.2-K76.4, K76.8, K76.9, Z94.4 |
| Diabetes without complications | 1 | E10.0, E10.1, E10.6, E10.8, E10.9, E11.0, E11.1, E11.6, E11.8, E11.9, E12.0, E12.1, E12.6, E12.8, E12.9, E13.0, E13.1, E13.6, E13.8, E13.9, E14.0, E14.1, E14.6, E14.8, E14.9 |
| Diabetes with end organ damage | 2 | E10.2-E10.5, E10.7, E11.2-E11.5, E11.7, E12.2-E12.5, E12.7, E13.2-E13.5, E13.7, E14.2-E14.5, E14.7 |
| Hemiplegia or paraplegia | 2 | G04.1, G11.4, G80.1, G80.2, G81.x, G82.x, G83.0-G83.4, G83.9 |
| Moderate to severe renal disease | 2 | I12.0, I13.1, N03.2-N03.7, N05.2-N05.7, N18.x, N19.x, N25.0, Z49.0-Z49.2, Z94.0, Z99.2 |
| Any tumor | 2 | C00.x-C26.x, C30.x-C34.x, C37.x-C41.x, C43.x, C45.x-C58.x, C60.x-C76.x, C81.x-C85.x, C88.x, C90.x-C97.x |
| Leukemia | 2 | C81.x-C85.x, C88.x, C90.x-C97.x |
| Lymphoma | 2 | C81.x-C85.x, C88.x |
| Moderate to severe liver disease | 3 | I85.0, I85.9, I86.4, I98.2, K70.4, K71.1, K72.1, K72.9, K76.5, K76.6, K76.7 |
| Metastatic solid tumor | 6 | C77.x-C80.x |
| AIDS | 6 | B20.x-B22.x, B24.x |
